# Failure of Sterne- and Pasteur-Like Strains of *Bacillus anthracis* to Replicate and Survive in the Urban Bluebottle Blow Fly *Calliphora vicina* under Laboratory Conditions

**DOI:** 10.1371/journal.pone.0083860

**Published:** 2014-01-02

**Authors:** Britta von Terzi, Peter C. B. Turnbull, Steve E. Bellan, Wolfgang Beyer

**Affiliations:** 1 University of Hohenheim, Institute of Environmental and Animal Hygiene, Stuttgart, Germany; 2 Center for Computational Biology and Bioinformatics, University of Texas at Austin, Austin, Texas, United States of America; Rockefeller University, United States of America

## Abstract

This study aimed to elucidate the bacteriological events occurring within the gut of *Calliphora vicina*, selected as the European representative of blow flies held responsible for the spread of anthrax during epidemics in certain parts of the world. Green-fluorescent-protein-carrying derivatives of *Bacillus anthracis* were used. These lacked either one of the virulence plasmids pXO1 and pXO2 and were infected, or not infected, with a worm intestine phage (Wip4) known to influence the phenotype and survival of the pathogen. Blood meals were prepared for the flies by inoculation of sheep blood with germinated and, in case of pXO2+ strains, encapsulated cells of the four *B. anthracis* strains. After being fed for 4 h an initial 10 flies were externally disinfected with peracetic acid to ensure subsequent quantitation representing ingested *B. anthracis* only. Following neutralization, they were crushed in sterile saline. Over each of the ensuing 7 to 10 days, 10 flies were removed and processed the same way. In the absence of Wip4, strains showed steady declines to undetectable in the total *B. anthracis* counts, within 7–9 days. With the phage infected strains, the falls in viable counts were significantly more rapid than in their uninfected counterparts. Spores were detectable in flies for longer periods than vegetative bacteria. In line with the findings in both biting and non-biting flies of early workers our results indicate that *B. anthracis* does not multiply in the guts of blow flies and survival is limited to a matter of days.

## Introduction

As reviewed extensively elsewhere [21] insects have been implicated in the transmission of anthrax since at least the late 1800s. Biting flies, particularly *Hippobosca* and *Tabanus* species, were considered important vectors in Africa and Asia with tabanids (horse flies) held responsible for spread during the massive epidemic in Zimbabwe in 1978-9 [5]. Non-biting blow flies (*Chrysomya spp*.) have also been implicated as the principal vector of anthrax in browsing wild herbivores in the Kruger National Park, South Africa [1], [6] and in the white-tailed deer in Northern America [10], by first feeding on the body fluids of anthrax carcasses and then depositing highly contaminated faeces or vomit on adjacent vegetation later consumed by browsers.

Additionally, many experimental studies in both large animals (horses, goats, caribou) and laboratory guinea pigs confirm the abilities of both genera of biting flies to transmit anthrax, albeit at low transmission rates and only when animals are exposed to flies within short time windows after their infectious blood meal [11], [12], [18], [19], [21].

As stated by Fasanella et al. [7], flies are well adapted for collecting pathogens with their profusion of fine hairs on proboscis and body that readily collect *Bacillus anthracis*-laden material from the carcasses of dead animals as well as infective body fluids which the insects suck up.

Of considerable interest to the ecology of *B. anthracis* and the epidemiology of anthrax have been the circumstances under which the organism can cycle outside the mammalian or avian host and the extent to which it may do so. One intermittently, but poorly, studied area in this regard has been the fate of *B. anthracis* in the guts of flies that have fed on the body fluids of animals that have died of anthrax.

Graham-Smith [8] fed spores or vegetative cells of *B. anthracis* to *Musca domestica* and *Calliphora erythrocephala*. After feeding vegetative cells, *B. anthracis* was recovered from legs and wings for up to 24 h, for up to 3 days from the gut and for up to 5 days from the crop. When fed spores, the *B. anthracis* could be recovered for up to 10 days from legs and wings and up to 7 days from gut and crop. There were no vegetative cells detectable by microscopy in the gut, leading to the conclusion that the spores did not germinate within the guts of the flies. In dry feces and regurgitated material, *B. anthracis* was detected for at least 20 days after feeding spores.

In the experiments of Kraneveld and Mansjoer [11], *B. anthracis* did not multiply in the gut of tabanids fed on infected guinea pigs. Analysis of excrement spots on sterile filter paper revealed declining numbers of vegetative and spore forms excreted over a period of up to 13 days until the last fly died but with some isolations still made at that time. Without detail on the methods used to establish it, de Vos [6] states that blowflies may be life-long carriers although vegetative *B. anthracis* disappear from their digestive tracts within two weeks of feeding on a carcass.

Recently Fasanella et al. [7] took a 3-pronged approach to assessing the fate of *B. anthracis* in the common housefly (*Musca domestica*). In vitro observations led them to conclude that the gut content of houseflies (*Musca domestica*) represents a favorable habitat for the germination and replication of anthrax spores taken up by flies feeding on an anthrax carcass.

The study reported here was designed to look for evidence of survival and multiplication within the guts of flies in vivo, in this case *Calliphora vicina* as a European representative of blow flies. The results indicated that multiplication did not occur in the guts of these flies and survival was limited to a matter of days. This is consistent with the hypothesis of Blackburn et al. [2] and Hugh-Jones and Blackburn [9] that the roles of necrophilic flies in the epidemiology of anthrax are confined to the immediate outbreak rather than being of long-term duration.

## Materials and Methods

### Strains

The following cultures were used, chosen for being able to be handled at BSL2. Strains A1 and A73 were derivatives, respectively, of the Sterne 34F2 vaccine strain (pXO1+/2−) and a Pasteur-like (pXO1−/2+) strain. Strains A1gfpWip4 and A73gfpWip4 are A1 and A73 lysogenized with Wip4, a worm intestine phage known to inhibit sporulation [17] (kindly provided by R. Schuch, Rockefeller University, New York, during a study visit at the University of Hohenheim's laboratories) and engineered to encode the green fluorescent protein (*gfp*) (plasmid pUTE610 kindly provided by T. Koehler, University of Texas, Houston).

### Spore suspensions

Spore suspensions of the four *B. anthracis* strains were prepared as published earlier [20]. Briefly, cultures were incubated on sporulation agar for ten days, the lawn harvested, washed, heat treated for 30 min at 65°C and finally resuspended in sterile 0.9% NaCl (saline) with 0.2% gelatine to concentrations of about 10^9^ spores/ml.

### Hatching of flies

Larvae on the 3rd larval stage were purchased from Rod's World, Esslingen, Germany, and held within a plastic cage under room temperature for about 3 days when they had pupated. Hatching occurred 11–13 days later. At this time the imagos were fed only water until the infection on day 16.

### Feeding the flies

**Figure 1:**
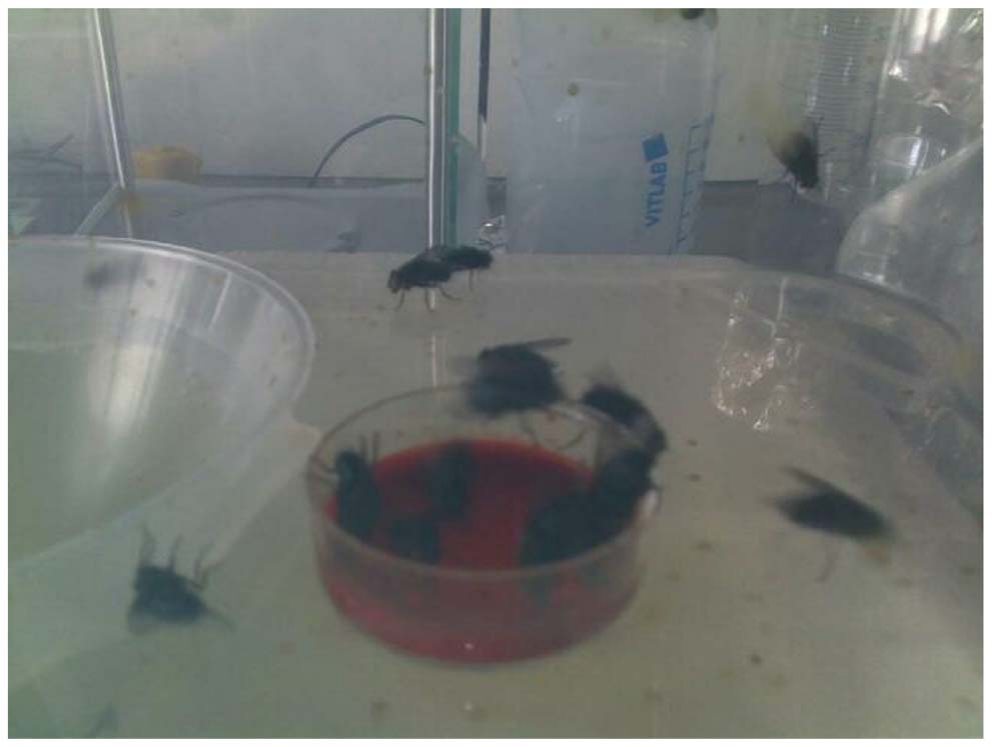
Feeding flies with the infected blood.

Spores (10^6^ per ml initial concentration) were germinated by incubation for 2.5 h at 36±1°C in brain-heart infusion broth (purchased from BD, #237500) supplemented with 1 mM L-alanine, and 5 µg/ml of erythromycin to sustain the gfp plasmid. For capsule formation the suspension was supplemented with 7% NaHCO_3_ and incubated under a 20% CO_2_ atmosphere. After microscopic confirmation of capsule formation, the cells were pelleted at 3500 x g for 15 min, and the pellet resuspended in 6 ml of defribinated sheep blood (Oxoid Deutschland GmbH, Wesel, Germany). The target bacterial concentration in the blood meals was 10^7^ cfu/ml. The blood was transferred to petri dishes which were placed in the fly boxes for 4 h (Fig. 1) The actual numbers of total viable cell counts (vegetative cells and spores) and spore counts were determined at the beginning and end of the 4-hour feeding time.

### Treatment of infected flies

After the 4 h feeding time, the flies were cooled to 4°C to immobilize them and 10 collected individually into separate tubes and killed by freezing at −80°C for 10 min. They were then externally disinfected by incubation in 1 ml of 0.1% peracetic acid for 1 h. After neutralization of the disinfectant by incubation in 1 ml of sterile saline containing 0.3% sodium thiosulfate for 30 min the flies were washed with sterile saline and the washing solution tested for sterility. Each fly was then transferred to 200 µl of fresh sterile saline and mechanically crushed. From this suspension the total and spore counts (i.e. pre- and post-heating at 65°C for 30 min) were determined on semi-selective trimethoprim-sulfamethoxazole-polymyxin blood agar (TSPBA) plates [21] via serial dilution counts. Thereafter, 10 flies a day up to 7 to 10 days were similarly caught, frozen and assayed. The feeding dish with non-infected feed (12 g wheatmeal and 40 g sugar dissolved in 40 ml water with heating and with 10 ml of apple puree added) was left in the cage until the end of the experiment, supplemented daily with sugar water.

### Microbiology

Undiluted and tenfold dilutions were plated on duplicate TSPBA plates before and after heating at 65°C for 15 min. After overnight incubation at 36±1°C total and spore counts were determined with confirmation of identities of the colonies done on the basis of morphology and expression of gfp. Where applicable the presence of phage Wip4 was confirmed by PCR using the published primers [17].

### Statistical Analysis

The data were analyzed by fitting both total cell counts and spore counts to independent variables including time since fed infected bloodmeal, *Bacillus anthracis* strain cell type, and presence of Wip4 using Generalized Estimating Equations (GEEs) with an independent correlation structure [22]. The goal of this analysis was to determine how total cell and spore counts in flies change over time after being fed an infectious bloodmeal, and whether strain types or presence of Wip4 increase or decrease the rate of decline. Based on exploratory analyses of model residuals, the outcome (total cell counts or spore counts) was transformed using the log (X +1) transformation and then modeled with GEEs using a log-link and with the variance modeled as a quasipoisson. The best model was chosen amongst 5 pre-specified models (which included the most saturated model including all variables and a 4-way interaction between them) using Quasilikelihood Information Criterion (QIC). Finally, while model selection allows us to select which variables should be included in the model, it does not directly allow inference on how each treatment group compares with each other. Thus, we conducted *post hoc* pairwise comparisons between treatment groups by using the bootstrap procedure (where we bootstrapped within the level of independence—each treatment-group-day combination) and the best model chosen above to create confidence intervals on the difference between rates of spore and cell decline in pairs of treatment groups.

## Results

A comparison of pre- and post-heating cell counts from the bloodmeal before and after the 4 h feeding period indicated that the majority of the cells taken up by flies were in the vegetative form (Table 1).

**Table 1:** Total viable (pre-heating) and spore (post 65°C for 30 min) counts in the blood meal.

	Before feeding		After 4 hours feeding	
	total cell count (cfu) per ml	spore count/in % of total cfu per ml	total cell count (cfu) per ml	spore count/in % of total cfu per ml
**A1gfp**	3.5×10^7^	4.9×10^5^/1.4%	1.3×10^8^	5,5×10^5^/0.4%
**A1gfpWip4**	7.9×10^6^	7.0×10^3^/0.09%	2.1×10^7^	5,0×10^3^/0.02%
**A73gfp**	4.0×10^7^	8.7×10^4/^0.22%	6.5×10^7^	1.7×10^5^/0.26%
**A73gfpWip4**	5.3×10^6^	7.7×10^4^/1.45%	1.1×10^7^	4×10^2^/0.004%

**Figure 2:**
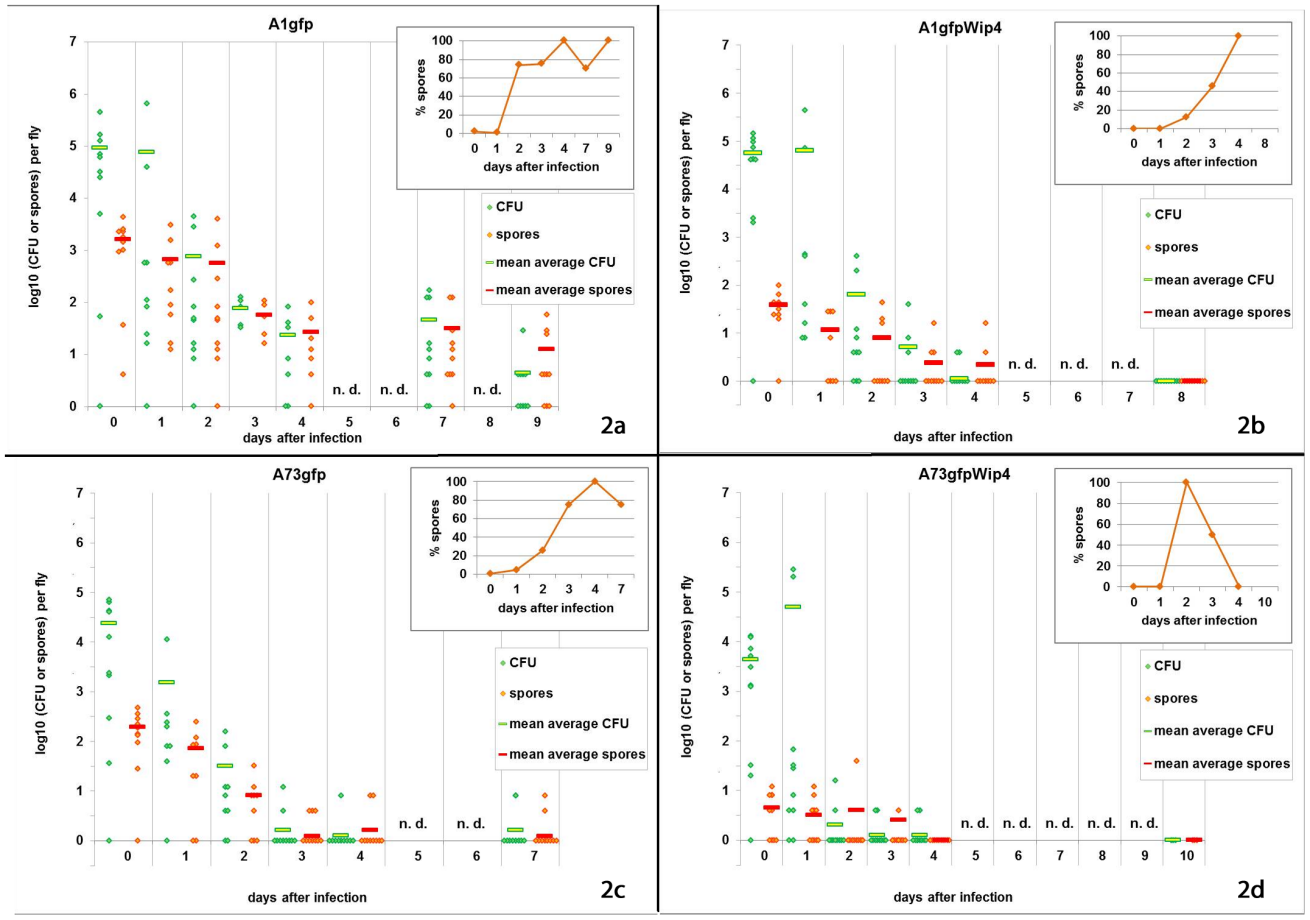
Determination of cell counts of inner organs of flies. Each dot indicates a single fly. n.d. – not done

In the absence of Wip4, strains A1gfp and A73gfp both showed steady declines in the total *B. anthracis* counts from initial mean levels of, respectively, 10^5^ and >10^4^ per fly steadily to <10 bacteria on days 9 and after day 4 respectively (Figs 2a, 2c). In both cases, the proportion of the counts which were in spore form increased from negligible to nearly 100% of all counted cells by day 4. In the case of A1gfp, on day 9, spores were still detected in 7 of the 10 flies tested. With A73gfp, on days 4 and 7 (after which there were no further flies) the spores were only detected in 2 out of ten flies, the remainder being negative.

**Figure 3:**
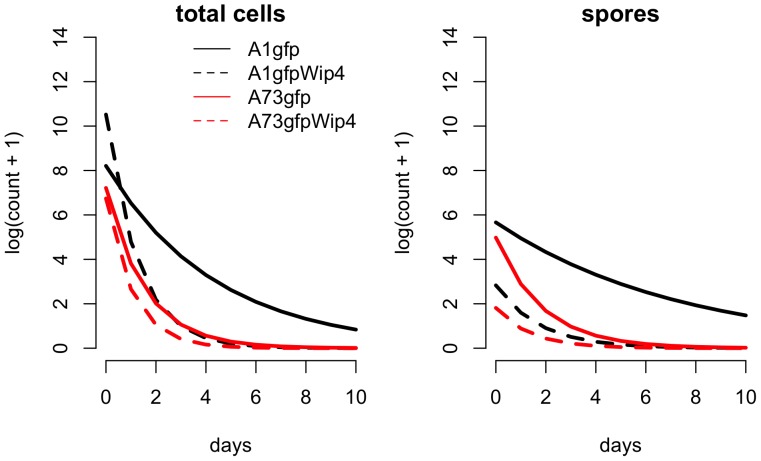
Lines show the best fitting Generalized Estimating equation Model (**Table 1**) for each strain-phage combination for both total cell counts and spores.

With the phage-infected strains, the falls in viable counts were significantly more rapid than their uninfected counterparts (Fig 2b, 2d, 3 and table S1). A1gfpWip4 and A73gfpWip4 both showed steady declines in the total *B. anthracis* counts from initial mean levels of, respectively, 2×10^4^ and 9×10^2^ per fly steadily to <10 bacteria on day 2. With A1gfpWip4, on day 3, 7 of the 10 flies were negative. On day 8, viable *B. anthracis* was not found. The proportions of cell counts that were spores increased from <1% at the first examination to nearly 100% on day 4. In the case of strain A73gfpWip4, by day 2, 8 of the 10 flies were negative and the proportion of spores in the other two was 100%. Re-isolates were checked by PCR for the presence of the Wip4 phage and were found to be positive regardless of the heat shock procedure. Colonial morphology did not reveal the presence or absence of the phage.

**Figure 4:**
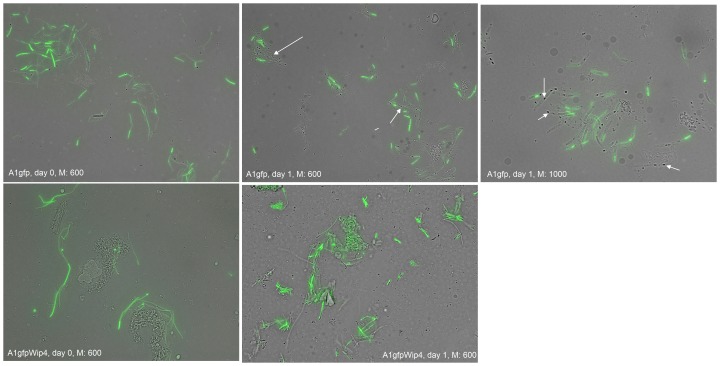
Crop content of one fly each at days 0 and 1after infection. M: Magnification, 60× or 100× oil immersion objectives. Arrows indicate spores.

To detect possible changes in the cell status of *B. anthracis* not clear from culture of heated vs. non-heated samples the cell status was also monitored by fluorescent microscopy, as illustrated in Fig. 4. Only in the crop of the flies analyzed on day 1 after infection from the group infected with strain A1gfp were sporulating cells visible. Notably, there were no sporulating cells visible in the Wip4 infected derivative of A1gfp.

Our model selection procedure chose one of the second most complicated models as the best-fitting model (Table 2). This model included all fixed effects and two three-way interactions, but not a full four-way interaction between all fixed effects. Therefore, there are statistically significant effects of phage presence and strain type on the rates of spore and cell count over time (Tables 2 and S1, Figure 3). Furthermore, as shown in Table S1 many of these effects were strong enough to be seen in significant differences (i.e., confidence intervals not including 0) in rates of decline and initial number of cells or spores (i.e., intercepts) between treatment groups as identified in *post hoc* pairwise comparisons.

**Table 2:** Model selection table.

model	formula	df	QIC	ΔQIC	quasilikelihood
**1**	cell type * day * phage + phage * strain * day	12	−828.9	0	426
**2**	cell type * phage * strain * day	16	–826.5	2.4	428.5
**3**	cell type * phage + phage * strain * day	10	–819.8	9.1	419.6
**4**	cell type * day + phage * strain * day	10	–796	32.9	408
**5**	cell type * day * strain + phage * strain * day	12	–795.6	33.3	409.9
**6**	cell type * strain + phage * strain * day	10	–779.1	49.8	399.6
**7**	cell type + phage * strain * day	9	–772.7	56.2	395.6
**8**	day * phage	4	–516.2	312.7	262.9
**9**	day * strain	4	–445.9	383	226.6
**10**	day * cell type	4	–358.2	470.7	183
**11**	day	2	–262.5	566.4	133.3

Generalized estimating equations with an exchangeable correlation structure and quasipoisson log-link were fit to log(counts+1) of spores and cells. The best model amongst the below competing model formulas was chosen using the Quasilikelihood Information Criteria (QIC). In the formulas, ‘*’ indicate an interaction and ‘df’ indicates the numbers of degrees of freedom. Only models with a QIC ≤2 greater than the best model should be considered along with the best model, thus model 1 is the single best fitting model.

## Discussion

In line with earlier findings of Graham-Smith [8], Morris [14], and Kraneveld and Manjsoer [11] that *B. anthracis* was unable to multiply in the guts of, respectively, *Musca domestica* and *Calliphora erythrocephala* and tabanid flies, our results indicate that this organism is not only unable to multiply but fails to survive for much beyond 7 to 9 days in the guts of the blow fly, *Calliphora vicina*. *C. vicina*, the bluebottle fly, is the predominant representative of blow flies in Europe. Our results also support the contention of de Vos [6] that vegetative *B. anthracis* disappear from the digestive tracts of blow flies (*Chrysomya albiceps* and *Chrysomya marginalis*) within two weeks of feeding on a carcass, but do not support his statement that blow flies may be life-long carriers.

Given the high number of viable *B. anthracis* found in our flies immediately after feeding on blood contaminated with vegetative cells, the hypothesis of Blackburn et al. [2] and Hugh-Jones and Blackburn [9] that necrophilic flies play a role as case multiplier if in direct connection to a carcass appears plausible. Fasanella et al. [7] observed a very rapid decline from originally high numbers of living cells in the spots of flies fed either on a carcass or on contaminated blood, as reported almost a century before by Morris [14] in biting flies. Together with our results these findings raise the question of how long the excrements of flies may be infective; this probably depends on whether either spores or vegetative cells are ingested and subsequently excreted.

Our finding that spore counts decline more slowly than that of vegetative cells agrees with Graham-Smith‘s [8] observation that *B. anthracis* was detectable in flies for longer periods when fed spores than when fed vegetative bacteria. This suggests that flies excrete infectious materials for longer durations after having ingested spores versus vegetative cells. Whether disseminated bacilli survive as infectious spores depends on their physiological status during deposition and, in case of vegetative cells being deposited, on the environmental conditions allowing or preventing sporulation [13]. It remains to be investigated how these factors influence the ecology under natural conditions.

Microscopy of individual flies (one each per day and group) showed that sporulating cells became visible on day 1 in the crop of a fly investigated from group A1gfp (strain 34F2) but not in a fly infected with A1gfpWip4 and not in either of the flies tested from groups A73gfp and A73gfpWip4. It should be noted that the total viable cell and spore counts in the blood meal before and after the 4 h feeding period indicate that the majority of the cells taken up by the flies were in the vegetative form, although substantial numbers of spores were also ingested. The relative spore numbers detected by culture from the flies at day 0 after infection were very similar to those counted in the blood meal immediately after the feeding period, again indicating that rapid and substantial sporulation of vegetative cells did not occur within the fly gut. Also, the spore counts in the fly guts declined from the outset, though at a significantly slower rate than the vegetative cell counts. The sporulating cells visible on day 1 in a fly infected with A1gfp may have been missed by culturing samples after heat treatment. While the source of spores isolated within the flies remains uncertain, the changes in vegetative cell and spore counts with time after feeding (Fig. 2 and 3) appear to indicate that sporulation did not occur within the flies or, as in the case of A1gfp, to a small extent which did not substantially add to the overall survival of *B. anthracis* in the flies.

Fasanella et al. [7] observed greater multiplication of cells in vitro after inoculation of spores into aseptically extracted gut contents from flies fed blood, as compared with a range of sugar, milk or egg diets, and interpreted this as indicating that the gut content of flies represents a favorable habitat for the germination and replication of anthrax spores taken up by flies feeding on an anthrax carcass. However, with houseflies fed on the carcass of a rabbit that had died of anthrax 36 h previously, concentrations of approximately 2.5 – 4×10^4^ cfu/ml were found in the vomit or excreted fly spots at 10 h, but numbers declined to undetectable at 24 h. Similarly, vegetative forms of *B. anthracis* found in gut contents aseptically removed from flies 2 h after feeding on a rabbit blood meal, inoculated with spores of *B. anthracis* and replaced every 30 min, was interpreted as in vivo germination within the guts of the flies. However, germination in animal blood starts irreversibly within minutes of inoculation of dormant spores leading to non-heat resistant spores [4]. Such spores, if taken up by flies, may develop into encapsulated vegetative cells which, as indicated by our results and previous data from the literature, do not replicate within the fly gut.

Recently Schuch and Fischetti [17] noted the ability of lysogenic phages to alter phenotypic features in *B. anthracis*, including blocking or promoting sporulation, inducing exopolysaccharide expression and biofilm formation and enabling long-term colonization of a soil environment and of the intestinal tract of an invertebrate, the redworm *Eisenia foetida*, by vegetative cells. Derived from “*B. anthracis*-like” (*B. cereus s. l.*) *E. foetida* gut bacteria, Wip4 was found to block sporulation of the Sterne vaccine strain 34F2 in vitro and to enable the survival of Wip4 lysogen of a Δ34F2 (strain cured of φ20) as a vegetative cell in soil and the worm gut [16].

In the present study the Wip4 infection did not confer an advantage for survival in the fly gut but rather led to a significantly faster decline in cell counts, which might have been a consequence of the induction of the phage followed by the lysis of the cells. The possible action of lytic phages in the fly gut could also be one explanation for flies tested negative for living *B. anthracis* even after feeding on fresh carcasses [15] or from seven out of eight pools of necrophilic flies collected from carcasses of red deers in West Texas [2]. Further studies, investigating the possibility of induction of lysogenic phages and their lytic replication under the physiological conditions of flies (or other vectors) fed on *B. anthracis* contaminated blood would be desirable.

Phages could also influence the number of living cells remaining in the spots deposited by defecation or regurgitation of necrophilic flies on the vegetation surrounding a carcass. Feces and vomits of necrophilic flies fed on a carcass have been shown to contain living cells of *B. anthracis*, at least for several hours [7] or days under laboratory conditions [3], [8], [14], as reviewed in Blackburn et al. [2] and Hugh-Jones and Blackburn [9]. Accordingly, large outbreaks in browsing herbivores have often been attributed to the spread of spores of *B. anthracis* by necrophilic flies to the vegetation in the close vicinity of a carcass [1], [10], [21], referred to as the case-multiplier-hypothesis [2]. However, the actual epidemiological role of this vector-born cycle has not been substantiated by quantitative data so far. Field studies, aimed at determining the number of living spores in the deposits of necrophilic flies on the vegetation in relation to (i) the status of the cells (spores vs. vegetative cells) taken up by flies feeding on carcasses of different age and condition, (ii) the number and status of cells surviving within the gut of different fly species, and (iii) the number and status of cells deposited on the vegetation and their fate in relation to environmental conditions [13] should be undertaken to finally verify the importance of this epidemiological cycle in nature. Similar quantitative investigations would also be desirable to substantiate the role of biting flies in the transmission of the disease.

## Supporting Information

Table S1
***Post hoc*** **comparisons between treatment groups.** By bootstrapping the data and refitting the best mode (Table 2), we calculated confidence intervals for differences in the intercept as well as the rate of cell count declines for all pairwise combinations of treatment groups, where each treatment group is modelled as 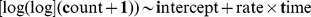. For instance, the top left cell indicates that the corresponding difference in rates: (rate A73gfpWip4 spores) – (rate A73 spores) = −0.17 with 95% confidence interval (−0.49 to 0.071).(DOCX)
