# Supplementary material for: Failure of Sterne- and Pasteur-Like Strains of Bacillus anthracis to Replicate and Survive in the Urban Bluebottle Blow Fly Calliphora vicina under Laboratory Conditions
Source: PLoS One. 2014 Jan 2;9(1):e83860. doi: 10.1371/journal.pone.0083860 (PMC3879289; doi:10.1371/journal.pone.0083860)
Supplement: Table S1 — Post hoc comparisons between treatment groups. By bootstrapping the data and refitting the best mode (Table 2), we calculated confidence intervals for differences in the intercept as well as the rate of cell count declines for all pairwise combinations of treatment groups, where each treatment group is modelled as . For instance, the top left cell indicates that the corresponding difference in rates: (rate A73gfpWip4 spores) – (rate A73 spores) = −0.17 with 95% confidence interval (−0.49 to 0.071). (DOCX) [file pone.0083860.s001.docx]

**Table S1: *Post hoc* comparisons between treatment groups.**

| Wip4+ vs Wip4- | | | | |
| --- | --- | --- | --- | --- |
|  | A73 spores | A1 spores | A73 total cells | A1 total cells |
| rate | -0.17 (-0.49, 0.071) | -0.44 (-0.7, -0.29) | -0.29 (-0.58, -0.027) | -0.56 (-0.75, -0.42) |
| intercept | -1 (-1.4, -0.66) | -0.69 (-0.99, -0.4) | -0.068 (-0.43, 0.28) | 0.25 (-0.048, 0.52) |
| total cell vs spores | | | | |
|  | Wip4- | Wip4+ |  |  |
| rate | -0.094 (-0.17, -0.02) | -0.22 (-0.43, 0.045) |  |  |
| intercept | 0.37 (0.16, 0.58) | 1.3 (1, 1.6) |  |  |
| A73 vs A1 | | | | |
|  | Wip4- | Wip4+ |  |  |
| rate | -0.41 (-0.57, -0.28) | -0.14 (-0.41, 0.092) |  |  |
| intercept | -0.13 (-0.37, 0.091) | -0.45 (-0.78, -0.14) |  |  |

By bootstrapping the data and refitting the best mode (Table 2), we calculated confidence intervals for differences in the intercept as well as the rate of cell count declines for all pairwise combinations of treatment groups, where each treatment group is modelled as $log(log \left( \mathrm{count}+1 \right)) \sim\mathrm{intercept}+rate\times time$. For instance, the top left cell indicates that the corresponding difference in rates: (rate A73gfpWip4 spores) – (rate A73 spores) = -0.17 with 95% confidence interval (-0.49 to 0.071).
